# Community-Based Measures for Mitigating the 2009 H1N1 Pandemic in China

**DOI:** 10.1371/journal.pone.0010911

**Published:** 2010-06-18

**Authors:** Sanyi Tang, Yanni Xiao, Youping Yang, Yicang Zhou, Jianhong Wu, Zhien Ma

**Affiliations:** 1 College of Mathematics and Information Science, Shaanxi Normal University, Xi'an, People's Republic of China; 2 Department of Applied Mathematics, Xi'an Jiaotong University, Xi'an, People's Republic of China; 3 Centre for Disease Modeling, York University, Toronto, Ontario, Canada; Duke University Medical Center, United States of America

## Abstract

Since the emergence of influenza A/H1N1 pandemic virus in March–April 2009, very stringent interventions including *Fengxiao* were implemented to prevent importation of infected cases and decelerate the disease spread in mainland China. The extent to which these measures have been effective remains elusive. We sought to investigate the effectiveness of *Fengxiao* that may inform policy decisions on improving community-based interventions for management of on-going outbreaks in China, in particular during the Spring Festival in mid-February 2010 when nationwide traveling will be substantially increased. We obtained data on initial laboratory-confirmed cases of H1N1 in the province of Shaanxi and used Markov-chain Monte-Carlo (MCMC) simulations to estimate the reproduction number. Given the estimates for the exposed and infectious periods of the novel H1N1 virus, we estimated a mean reproduction number of 1.68 (95% CI 1.45–1.92) and other A/H1N1 epidemiological parameters. Our results based on a spatially stratified population dynamical model show that the early implementation of *Fengxiao* can delay the epidemic peak significantly and prevent the disease spread to the general population but may also, if not implemented appropriately, cause more severe outbreak within universities/colleges, while late implementation of *Fengxiao* can achieve nothing more than no implementation. Strengthening local control strategies (quarantine and hygiene precaution) is much more effective in mitigating outbreaks and inhibiting the successive waves than implementing *Fengxiao*. Either strong mobility or high transport-related transmission rate during the Spring Festival holiday will not reverse the ongoing outbreak, but both will result in a large new wave. The findings suggest that *Fengxiao* and travel precautions should not be relaxed unless strict measures of quarantine, isolation, and hygiene precaution practices are put in place. Integration and prompt implementation of these interventions can significantly reduce the overall attack rate of pandemic outbreaks.

## Introduction

The 2009 influenza A/H1N1 pandemic outbreaks have exhibited some unique patterns in mainland China [1], [2]. The majority of reported H1N1 cases were initially diagnosed in coastal areas and urban structures such as Shanghai and Beijing, where case importation are most probable. In an attempt to contain the community-wide spread the disease, a series of stringent non-pharmaceutical interventions (NPIs) were rapidly implemented by the central government, including intensive contact tracing followed by quarantine of individuals suspected of being exposed to the disease, isolation of ill individuals with symptoms, and school closure. *Fengxiao*, a tightly monitored measure of movement restriction [3], [4], was also put in place to proscribe college and university students, faculty, and staff members to leave their campuses, and to disallow on-campus visits while maintaining essential services and normal scientific activities (File S1).

**Figure 1:**
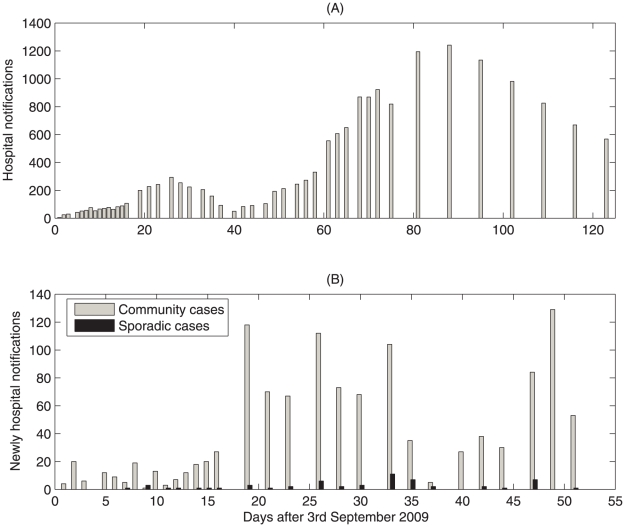
The reported cases of H1N1 influenza for the province of Shaanxi. (A) Non-regularly daily number of hospital notifications for the province of Shaanxi from September 3rd, 2009 to January 3rd, 2010; (B) Non-regularly daily reported community and sporadic cases from September 3rd to October 23rd 2009.

Although these NPIs may have contributed to reducing disease incidence, their effectiveness from the standpoint of public health policy remains undetermined. Various surveillance data suggest a significant increase in the number of H1N1 cases following the week-long October National Day holiday starting on October 1st (Figure 1), and it is therefore natural to expect a similar trend during the Spring Festival in mid-February of 2010, when massive traveling is expected for a large segment of the population. Whether the increased mobility during this holiday contributed to the increase of disease cases is unclear, but the Chinese Center for Disease Control and Prevention (CDC) has already recommended reduction of traveling during the Spring Festival. Should the current declining trend be reversed by the massive travel during the Spring Festival, the issue whether *Fengxiao*, or its variation, shall be useful for the semester after the Spring Festival must be addressed urgently and falls within the scope of this study. The epidemic curve in Figure 1 displays bimodality with a turning point around October 1st of 2009. Unlike the two epidemic waves (spring and fall waves) reported in many areas of the world, there was no significant weather change around the above turning point and the schools and universities/colleges already started a month ago. The A/H1N1 pathogen was reseeded in more general population with the beginning of the fall semester in late August and this reseeding certainly generated the initial growth of infection in Figure 1. This fall wave declined quickly due to very strict interventions in early September, but the declining trend was reversed following the October National Day holiday during which population mobility increased and *Fengxiao* was suspended. So, although the bimodality of an epidemic may in general be due to a variety of factors such as varying rates of mobility, exogenous seasonal process and/or endogenous changes in the population, it is natural we chose to focus here on the impact of mobility and NPIs on the pandemic infection.

To evaluate the effectiveness of NPIs considered here, we follow a modeling approach for stratification of the population [5] according to the clinical progression of disease and epidemiological status of the individuals (Figure S1). We parameterize the model using data obtained for the laboratory-confirmed cases of H1N1 in the province of Shaanxi, and estimate the reproduction number of disease transmission. In the context of a novel influenza virus invading a naive population, estimates of the reproduction number will determine the potential and severity of an outbreak, and provide critical information for identifying the type of disease interventions and the intensity of mitigation measures required for achieving maximum protection of community health. We evaluate various NPIs and extend the model to a meta-population framework with network structures to address the impact of *Fengxiao* and travel reduction on pandemic mitigation during and following the Spring Festival holiday.

## Results

### Model-based estimates

We employed an adaptive Metropolis-Hastings (M-H) algorithm to carry out extensive Markov-chain Monte-Carlo (MCMC) simulations [6], and to estimate mean values of parameters including quarantine rate, relative infectiousness of the pre-symptomatic class, isolation rate, the reproduction number of disease transmission and the initial data. Using large sample realization, we fit the model to data of hospital notification for H1N1 cases on the bases of exponential growth during the early stages of the outbreak (see Materials and Methods and File S1 Appendix B). Given the model structure with quarantine and isolation (Figure S1), the use of next generation matrix [7], [8] yields an expression for the control reproduction number when control measures are in force as
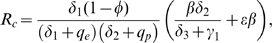
where 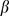 is the baseline transmission rate; 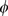 is the proportion of the exposed individuals who performed effective precaution; 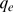 and 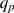 model the rate of quarantining infected but not yet infectious and infectious pre-symptomatic individuals respectively; 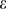 is the relative transmissibility of pre-symptomatic infection; 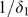 and 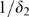 are respectively the durations of latency and infectiousness before the onset of symptoms; 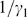 is the duration of infectiousness following symptoms onset; and 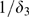 is the period of time spent in symptomatic stage before isolation (hospitalization). The epidemiological interpretation of the above formula term-by-term is given in File S1 Appendix A.

Based on recently available estimates of the epidemiological characteristics such as the incubation and latent periods, and the duration of treated (untreated) symptomatic infection (see Table S1), and based on our model 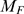 ignoring asymptomatic infection (see File S1 Appendix A) we estimated the mean control reproductive number (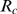) as 1.682 (95% CI 1.446–1.918, Figure S2 (A)), the mean quarantine rate 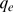 as 0.125 and 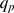 as 0.387 for the period from September 3rd to September 21st 2009. The number of individuals who were exposed to the virus was also estimated to be 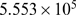 with large variation, and the initial values for other compartments were listed in Table 1.

**Table 1:** Parameter estimates for the 2009 H1N1 influenza in the province of Shaanxi, China.

Parameter	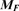			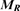			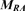		
	Mean	Std	Geweke	Mean	Std	Geweke	Mean	Std	Geweke
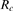	1.682	0.118	0.974	1.679	0.126	0.987	1.792	0.125	0.976
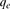	0.125	0.108	0.878	0.135	0.122	0.875	0.061	0.075	0.827
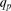	0.387	0.281	0.966	0.381	0.279	0.972	0.123	0.111	0.998
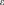	0.515	0.287	0.941	0.505	0.289	0.957	0.556	0.286	0.914
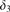	1.094	0.433	0.923	1.079	0.435	0.997	1.164	0.444	0.873
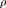	–	–	–	–	–	–	0.802	0.187	0.893
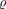	–	–	–	–	–	–	0.433	0.273	0.808
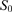	555310	261030	0.935	–	–	–	–	–	–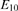	8	6	0.954	9	6	0.929	28	24	0.812
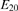	4	3	0.916	4	3	0.983	17	20	0.784
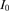	5	4	0.899	4	3	0.857	6	6	0.663
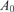	–	–	–	–	–	–	36	28	0.747
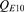	87	9	0.989	87	9	0.983	86	10	0.980
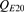	6	5	0.970	6	5	0.951	6	5	0.963

To get rid of effect of estimating initial susceptible populations on the estimates, we also used a simplified version of our baseline model, model 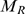 (see File S1 Appendix A), which is suitable to fit the model with the initial exponential growth of the outbreak. This yields the mean control reproductive number as 1.679 (95% CI 1.427–1.931, Figure S2 (B)), the mean estimated quarantine rate 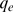 and 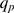 as 0.135 and 0.381, which agrees well with the above estimated 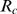 using a more complicated model. We also plotted the best-fit solution (Figure S3) and calculated R-square (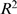) statistic to show goodness of fit, where the R-square value is 0.960. Density estimates of posterior densities of parameters and initial values based on model 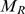 were shown in Figure S4. To assess the effect of asymptomatic infection on the control reproduction number 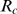, we repeated the above procedure but incorporating the asymptomatic (infectious) compartment into the 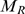 model (see 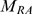, File S1 Appendix A). We obtained that the estimated control reproduction number is 2.184 (95% CI 1.819–2.549, Figure S2 (C)). This shows that including asymptomatic cases into the analysis leads to a greater control reproduction number with larger variation.

### Sensitivity analyses

**Figure 2:**
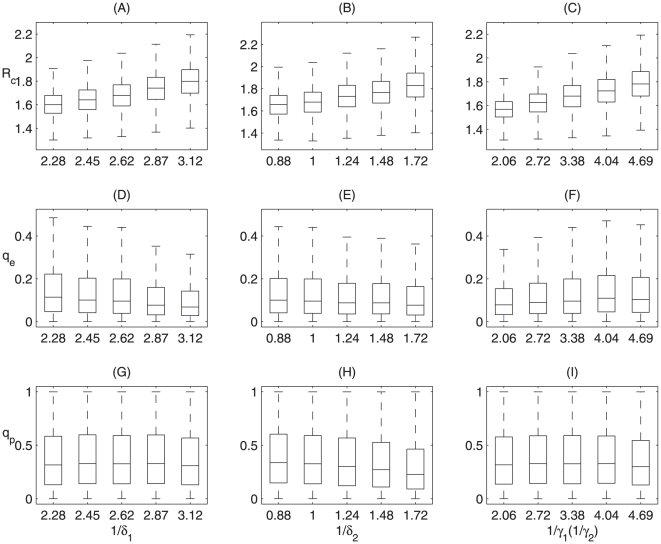
Sensitivity of the reproduction number and quarantine rate on model parameters. Sensitivity analyses for the effect of durations of latency and infectiousness before the onset of symptoms, and duration of infectiousness following symptoms onset on 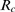 (A, B, C); on the fraction of exposed individuals who are quarantined during the latency (D, E, F) and pre-symptomatic infection (G, H, I), on the basis of model 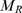.

To examine the effect of parameter changes (the ranges are given in Table S1), especially durations of latency and infectiousness before the onset of symptoms, and duration of infectiousness following symptoms onset, on the control reproduction number, quarantine rates 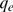 and 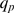, we carried out sensitivity analyses (Figure 2), using MCMC method. It follows that decreasing duration of latency results in a decline in 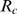 and an increase in quarantine rate 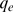, and the quarantine rate 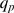 is not sensitive to variation of the latency (Figure 2 (A, D, G)). Decreasing the duration of infectiousness before the onset of symptoms results in a decline in 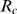, a slight increase in quarantine rates 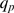 and 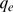 (Figure 2 (B, E, H)). Decreasing the duration of infectiousness following symptoms onset leads to a decline in 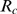, a decrease in quarantine rate 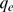, and little change of the quarantine rate 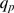 (Figure 2 (C, F, I)). The effect of varying these parameters (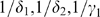 and 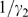) on estimates of the relative infectiousness of the pre-symptomatic class (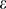) and the isolation rate (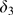) is shown in Figure S5 and our analysis suggests that parameters 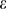 and 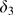 are not sensitive to variation of those four parameters.

**Figure 3:**
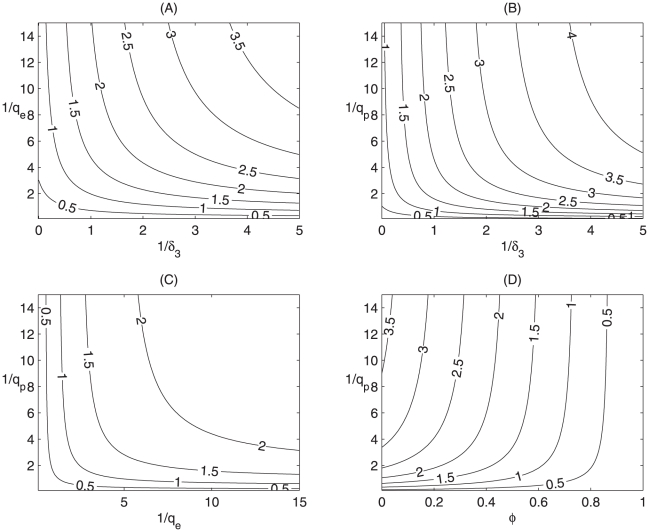
Contour plots of 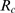. Plot contours of 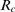 versus the average days to isolation 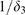 and average days from exposure to quarantine during (A) latency (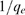), (B) pre-symptomatic infection (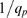); Plot contours of 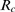 versus mean period from exposure to quarantine during pre-symptomatic infection 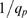 and (C) during latency 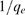, (D) hygiene precaution rate 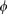. All other parameter values are as shown in Table S1.

The contour plots of Figure 3 (A,B) show the dependence of 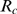 on average days from exposure to quarantine during latency 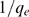 (or during pre-symptomatic infection 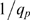) and average days from onset of symptoms to isolation 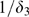. In both cases, the contour plots show that the control strategies (increasing 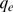 (or 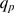) and 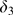) reduces the control reproduction number 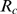, and hence A/H1N1 cases. With baseline parameter values (listed in Table S1 for model 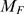), Figure 3 (A) shows that 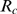 decreases from 2.233 (or 2.333) to 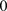 as 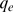 (or 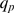) increases from 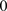 to 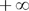, and the outbreak can be controlled when 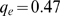 (or 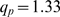) (corresponding to 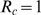). The contour plots of Figure 3 (C) shows that the outbreak could be controlled by either quarantining the exposed but not yet infectious individuals or isolating the infectious during their pre-symptomatic stages, as long as the measure was strict: the quarantine rate 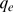 (or 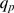) must be at the level of 0.801 or 2.1 for 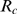 to fall below the unity. It follows from Figure 3 (D) that for a combination of quarantine and hygiene precaution measures, increasing 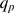 by 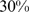 (while holding 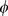 at the current level) can reduce 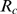 by 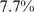, and increasing 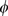 by 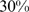 while holding the current quarantine level can reduce 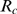 by 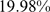.

### The Effectiveness of *Fengxiao*

To examine the effect of *Fengxiao* on controlling the spread among university campuses and between the campus and community, we formulate a metapopulation model, 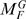, using the approach of [9] to couple the 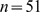 colleges and universities in Xi'an, the capital city of Shaanxi [10] together. The disease progression in each patch (campus) is tracked using the baseline model, and the transmission among patches is represented by the mobility of (untraced, unquarantined and unisolated) individuals from one patch to another on a given dispersal network (see S1 Appendix C for details). *Fengxiao* obviously can not be sustained for too long, and thus some decisions must be made for when this should be initiated, when it should be suspended, and how it should be integrated into other local control measures such as quarantine and precaution measures. Once the daily number of laboratory-confirmed cases in a given university 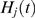 reaches a given threshold value 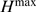, interventions involving *Fengxiao* or enhanced local control measures (quarantine and hygiene precaution) or a combination of both are implemented in the university. When the daily number of laboratory-confirmed cases in that university decreases to a low threshold value 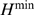, these interventions are suspended or weakened.

**Figure 4:**
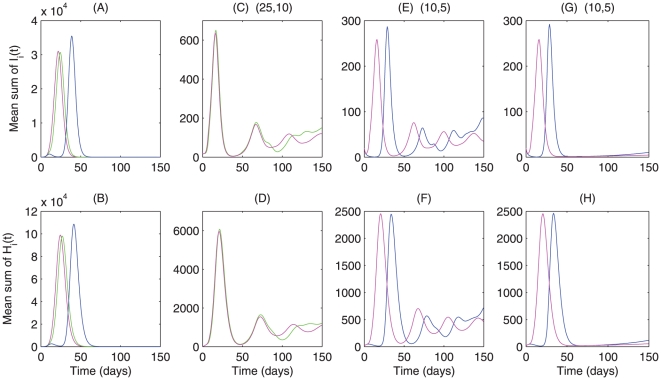
Effects of local quarantine, hygiene precaution and *Fengxiao* on the A/H1N1 influenza outbreaks in the province of Shaanxi. Numerical integrations of model 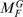 are given in S1 Appendix D. 500 independent simulations are carried out. Mean sum of 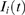 and mean sum of 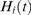 are plotted by employing (A–B) case 1, magenta curve without *Fengxiao*, green curve with 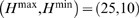 and blue curve with 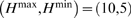; (C–D) case 2a (3a), magenta (green) curve with 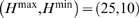; (E–F) case 2a (3a), magenta (blue) curve with 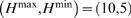; (G–H) case 2b (3b), magenta (blue)curve with 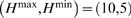. We fix the small world network structure as dispersal pattern.

Figure 4 (A–B) shows that *Fengxiao*, if implemented early, can delay the epidemic peak significantly and prevent the disease spread to the general population, though it may cause more severe outbreak due to the aggregate outbreaks within the universities/college. Late implementation of *Fengxiao* has little effect on the outbreak, with disease control outcomes similar to the case of no implementation of *Fengxiao*. To examine the interaction of other local control strategies and *Fengxiao*, we consider the scenario when (a) strengthening quarantine and hygiene precaution measures are taken without *Fengxiao* (Figure 4 (C–F) pink curves) or with *Fengxiao* (Figure 4 (C–F) green or blue curves) if 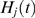 reaches 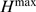; and (b) *Fengxiao* is suspended and other local control strategies are reduced to a low level when 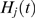 reduces the 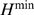. Simulations show, as indicated by Figure 4 (C–F), that magnitudes of the outbreaks become weaker and weaker as *Fengxiao* and strengthening control measures are switched on and off, and the sooner the local control measures are the less severe the outbreaks. Local control strategies affect the peak magnitudes while *Fengxiao* influences the peak timing. In general, comparing the green curve in Figure 4 (A–B) (*Fengxiao* only) with the pink curve in Figure 4 (C–D and E–F) (local control measure only) implies that strengthening local strategies is much more effective to mitigate outbreaks than implementing *Fengxiao*. To illustrate the impact of persistent local control strategies, we also simulate the scenario that local control strategies are switched back to a relatively high level when 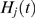 decreases to the threshold 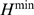 (Figure 4 (G–H)), and our simulations show that the relatively strong local control measures implemented when *Fengxiao* is suspended can greatly inhibit the successive wave.

### Effect of increasing travel during the Spring Festival

**Figure 5:**
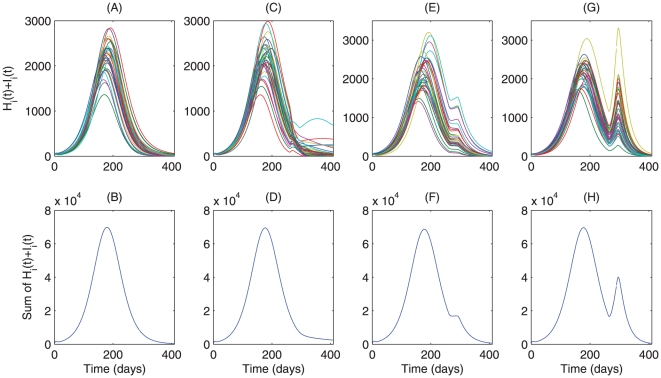
Effect of nationwide travel on A/H1N1 influenza outbreaks. We fix the small world network as dispersal pattern. Numerical integrations of model 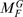 given in Appendix D(case 4) are employed. 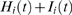 and summation of 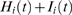 for all 32 patches in a single simulation are plotted for the model 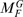 (A–B) with weak dispersal; (C–D) with large dispersal after day 260; (E–F) with a doubled susceptible population after day 260; (G–H) with a reduction of 20% local quarantine rates 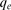 and 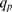.

To assess the impact of increasing nationwide travel or social activities during the Spring Festival on the immediate future trend of pandemic in mainland China, we use the aforementioned patch model with 32 patches (mainland China has 23 provinces, 4 municipalities and 5 autonomous regions) and conduct some simulations based on the parameter values for the province of Shaanxi. We consider scenarios with increasing the population mobility among the patches, weakening local control strategies or increasing susceptible populations in the period of late January to late February. Figure 5 (A–B) shows that the epidemic will continue to recede if nothing happens, and Figure 5 (C–D) shows that very strong spatial dispersal after day 260 (about February 1st) slightly increases the pandemic infection in some patches but does not change the downward trend overall. An immediate consequence of the nationwide travel and increased social activities is the increase of susceptible populations, and Figure 5 (E–F) shows that doubling the susceptible population in each patch will generate a quite spatially aggregated patterns of infections: a new large wave in some patches but a quite small wave in others. Weakening local control measures (quarantine or hygiene precaution measures) by decreasing 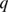 or 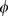 yields similar outcomes as increasing susceptible populations, except the new wave is much more significant (Figure 5 (G–H)).

**Figure 6:**
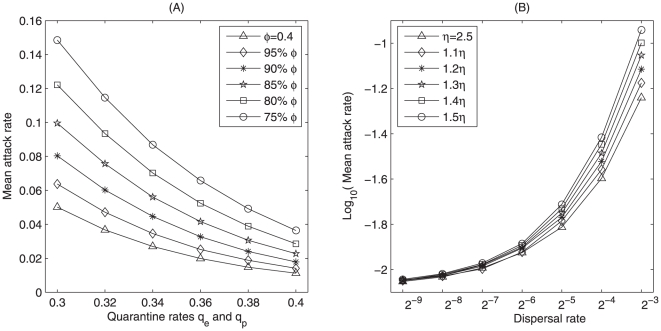
Effect of local control measures and nationwide travel on mean attack rate. We fix the small world network as dispersal pattern. 200 independent simulations are carried out by employing Appendix D(case 4). Mean attack rates versus (A) quarantine rates and hygiene precaution rate for the model 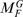; (B) dispersal rate and transport-related transmission rate for the model 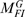.

Further, we calculate the variation of the attract rate in the period of late January to late June of 2010 due to weakening local control measures during the Spring Festival (late January to late February, 2010). Figure 6 (A) shows that weakening quarantine 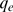 and 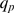 by 25% from 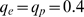 while holding the current hygiene precaution level (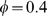) can increase the mean attack rate by 349% (95% CI 278%–420%), and decreasing 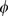 by 25% from 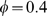 while holding the current quarantine level (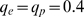) can increase mean attack rate by 225% (95% CI 177%–273%). It also shows that reducing both quarantine rates 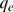 and 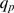 and hygiene precaution level by 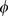 25% from 0.4 can increase mean attack rate by 1230% (95% CI 1064%–1396%). As the quarantine (or precaution) level decreases, the effect of hygiene precaution (or quarantine) becomes more pronounced in increasing attack rate.

**Figure 7:**
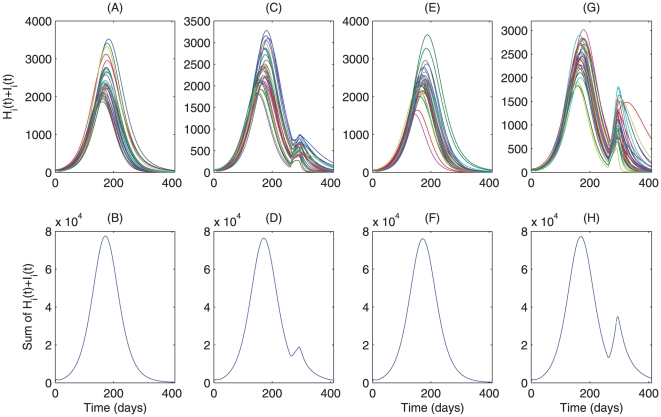
Effect of nationwide travel and infection during travel on A/H1N1 influenza outbreaks. We fix the small world network as dispersal pattern. Numerical integrations of model 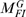 given in Appendix D(case 4) are employed. 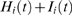 and summation of 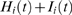 for all 32 patches in a single simulation are plotted for the model 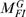 (A–B) with weak dispersal and low transmission during travel; (C–D) with large dispersal after day 260; (E–F) with high transport-related transmission rate after day 260; (G–H) with both large dispersal and high transport-related transmission after day 260.

Massive travel without significant improvement of the transportation system will likely increase the infection during the travel. To see the impact of this infection during the travel on the global outcome, we extended the full model 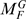 to incorporate the transport-related infection, see 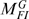 of the S1 Appendix C. Figure 7 (A–B) shows the receding epidemic with relatively weak mobility and low transport-related infection, while Figure 7 (C–D) shows that with strong mobility only after day 260 (about February 1st of 2010) the infection in some patches increases and causes a small new wave. Whilst high transport-related transmission rates with weak mobility hardly influence the infection (Figure 7 (E–F)), strong mobility coupled with high transport-related transmission rates will result in a new wave in many patches and cause a large new wave in most patches.

Similarly, we calculate the variation of the attract rate in the period between the late January and late June of 2010 due to massive travel and transport-related transmission during the Spring Festival. Figure 6 (B) shows that increasing transport-related transmission rate while holding dispersal at its current level has little impact on the mean attack rate, which confirms the results obtained in Figure 7 (E–F). It also shows that increasing dispersal rate from 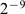 to 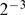 while holding the current transport-related transmission (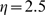) will increase mean attack rate by 552% (95% CI 398%–706%). Further, increasing both dispersal rate from 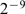 to 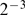 and transport-related transmission rate 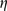 1.5 times its current level can increase the mean attack rate by 1198% (95% CI 944%–1452%). As the dispersal rate increases, the effect of transport-related transmission becomes more pronounced in increasing the mean attack rate.

## Discussion

Using initial data on the laboratory-confirmed cases of pandemic H1N1 influenza in the province of Shaanxi, we obtained estimates of the (control) reproduction number, NPIs parameter values and the number of individuals who were exposed to the virus. These estimates, along with information on population susceptibility, provided information about the utility or success of different community mitigation strategies. Our estimated reproduction number from the hospital notifications from September 3rd to September 21st is 1.682 (95% CI 1.446–1.918). This number seems to be higher than those obtained in published study of the data from Canada (1.25 to 1.38) [11] and lower than those from New Zealand (1.96, 95% CI 1.80–2.15) [12] during the Southern Hemisphere winter. Our estimate is more consistent with those from Mexico (1.2 to 1.6) [13] and from United States (1.7–1.8 after adjustment for increasing ascertainment of cases) [14]. We believe our estimation reflects the characteristics of the early epidemic in the province of Shaanxi, since the initial infection was introduced by students returning to universities at the beginning of the autumn term and secondary infections were generated through close contact of small clusters within the same dormitories or classes. Our estimation of the total populations exposed to the virus added further confirmation for this observation. Our estimation suggested that the population exposed to the virus is much lower than the total population of the province of Shaanxi, an indication of the effectiveness of NPIs such as quarantine, hygiene precaution measures and early *Fengxiao*.

Note that our estimation of the reproduction number may change slightly depending on the period chosen (see Appendix Table S2), and estimations using simplified models ignoring asymptotic infections yield similar estimates. Note that including asymptomatic infection either increases or decreases the control reproduction number 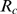, depending on the sign of 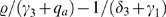 (the relative infectiousness of the asymptomatic infection versus infectiousness of the symptomatic infection) [15]. Our estimates result in the reproduction number with asymptomatic infection being greater than that in the absence of asymptotic infections. Therefore, the estimates without considering asymptomatic infection in our case may underestimate the disease spread. We note that the study [16] shows that assuming exponentially distributed latent and infectious periods always results in underestimating the reproduction number of an infection from outbreak data. As such, the control reproduction number, based on our data and model assumption of exponentially distributed latent and infectious periods, is underestimated.

*Fengxiao* as a public health policy in China, if implemented early, has the advantage of delaying outbreak peaks and thus provides the critically needed response time (e.g., vaccine production and facility preparation) but it can not prevent the eventual outbreak. *Fengxiao*, if not appropriately managed, may cause more severe outbreak due to the aggregate outbreaks within the universities/college while preventing the disease spread to the general population. It is important to remark that *Fengxiao* in mainland China is almost opposite to the school closure measure in other countries [17], [18]. Our findings are relevant to the conclusion by Cauchemez et al. that school closure might lead to reduction in the peak incidence of cases conditional upon children being sufficiently isolated or policy being well implemented [18].

Our analyses suggest that *Fengxiao* alone is not enough to mitigate pandemic infection but local control measures are capable of significantly inhibiting the successive waves so long as they remain in effect persistently. Comparison Figure 4 (E–F) and (G–H) implies that the occurrence of the second wave is more likely associated with the relaxation of interventions when *Fengxiao* is suspended, a similar situation was observed in the 1918 influenza pandemic in U.S. [19]. This observation indicates the potential risk of a new wave as people maybe less cautious of social distancing during the upcoming Spring Festival, and this new wave may require strong intervention strategies at its earlier stage since it will likely coincide with the beginning of the new academic term.

Massive transportation and social gathering during the Spring Festival could lead to relaxing local control measures and increasing the number of individuals exposed to the virus, both will likely generate a sizable new wave in some provinces. So there exists a strong correlation between massive travel and H1N1 outbreak, as obtained by Khan et al. [20]. The additional risk of transportation related infection and the massive home coming of farmers to villages from major cities will make the rural areas particularly vulnerable for the brunt of a renewed outbreak. Therefore, disease prevention and control measures must remain rigorous, as indicated by the Chinese Health Ministry [21]. The simulation results (Figure 6) show that increasing hygiene precaution or quarantine rate or both by 33.3% from 0.4 can respectively reduce the attack rate by 69% (95% CI 65%–74%), 78% (95% CI 74%–82%), and 93% (95% CI 92%–94%), respectively. Reducing dispersal 50% from 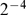 or transport-related transmission 33.3% from 3.75 or both could lead to a decline in attack rate by 56% (95% CI 44%–68%), 50% (95% CI 38%–62%) and 78% (95% CI 72%–84%), respectively.

The implications of our simulation results should be interpreted with care. Note that this study is a bit geographically specific, since the data for the province of Shaanxi used here is more complete and regular than those for the mainland China, but we hope the approaches we used are able to be applied more generally. Our findings suggest that early and comprehensive NPIs including *Fengxiao* are credited for the small epidemics so far, but how “early” is quite an optimal issue. That is, the decision for the trigger for *Fengxiao* and its suspension is crucial [22], [23]. This work was conducted before the Spring Festival and it would be natural to compare our simulated scenarios with the real situation. Unfortunately, as millions of Chinese complete one of the largest human migration through a complicated national network of transportation, reliable data about the dispersal rates and the transport-related infection is not available. We, thus, pseudorandomly generated dispersal rates independently and identically distributed among all patches for simulating the meta-population model. Our simulation results indicate the importance of reducing transportation related infection. But without reliable travel data during the Spring Festival, our simulations remain more qualitative. Despite these caveats, our simulation results strongly suggest prompt implementation of multiple NPIs is required to reduce a potential risk of new outbreak and to mitigate the new outbreak should it occur.

## Materials and Methods

### Data

We obtained the data on laboratory-confirmed cases of pandemic H1N1 influenza in the province of Shaanxi, China, from the province's Public Health Information System [2]. Data information includes the cumulative number of reported, the cumulative number of cured and the number of new cases (identified within two days or a week). The data were released and analyzed anonymously. There were 21 imported confirmed cases before August 31st, which did not generate secondary cases and hence were not included in our data set. On September 3rd, local cases were found and reported in the Xi'an Institute of Art and Science. Shaanxi Bureau of Health started to report cases daily then changed, on September 19th and November 17th respectively, to report once every two days and once every week. No data is available on weekends (Figure 1 (A)). All confirmed cases in mainland China were isolated in health care facilities with treatments and were assumed to be unable to infect others. Majority cases in the province of Shaanxi in early September were associated with university/college campuses (Figure 1 (B)) (reflected by the number of community cases vs the number of sporadic cases). After the National Day holiday season, there seemed to be no significant difference between the community cases and sporadic cases, and consequently after October 23 the numbers of community and sporadic cases have no longer been reported separately. According to a byelaw on pandemic A/H1N1 influenza, the Ministry of Health required that all medical institutes should directly report within 2 hours through national epidemic disease information system once they found cases which comply with the definitions of probable, clinical and laboratory-confirmed cases. So, we believe the data on laboratory-confirmed cases of pandemic A/H1N1 influenza from September to November were quite accurate [24]. Since December, however, any patient with fever did not have to have a confirmation for A/H1N1 virus for medical treatment, and hence H1N1 cases were under-reported since then.

### The model

We formulated a baseline model that reflects some key epidemiological properties of the pandemic A/H1N1 influenza and the implemented public health interventions (quarantine, isolation and hygiene precaution). The underlying structure of the model comprises of classes of individuals that are susceptible (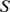), exposed but not yet infectious (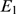), infectious but not yet symptomatic (pre-symptomatic) (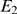) [22], infectious with symptoms (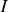), and recovered (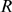). We considered quarantine of individuals that have been exposed to indexed cases (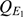 and 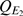), and further isolation and hospitalization of those who develop symptomatic infection during the period of quarantine (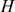). Our model also incorporates precautionary measures: when effective precautionary measures are taken, a proportion, 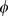, of the individuals exposed to the virus is protected from the infection. It is also assumed that those who were quarantined and/or isolated/hospitalized did not contribute to the spread. See Figure S1 for a schematic illustration of the baseline model, and Appendix Table S1 for parameter definitions. The model equations are given in S1 Appendix A. In order to investigate the effect of Fengxiao and travel reduction on pandemic mitigation, we extended the baseline model to a meta-population model with network structures. Dispersal rates are the same order as the total number of individuals in a patch on the basis of model formulation, resulting in the possibility where some patches may have small population sizes, and hence demographic stochasticity may become important in several ways. Sufficiently small dispersal rates as chosen as below, however, did not yield any patch with small population size in both the reality and our simulations. Therefore, we formulate our meta-population model deterministically. Recent studies such as [25] suggested that people born before 1950 might have residual immunity against the influenza A/H1N1 subtype (both seasonal and pandemic), it would be desirable to consider the impact of age structure. Our data on laboratory-confirmed cases from the province's public health information system [2], however, do not include information on age distribution. Consequently, we formulated the compartment model without incorporating age structure. Note that the susceptible population in our model is not the total population of the province, but rather the population (effectively) exposed to the virus. This population size is estimated from our model simulations.

### Simulations

Due to the non-regularly daily reported data from province of Shaanxi such as reporting delays on weekends and reporting policy changed, the daily hospital cases were generated by using the cubic spline interpolation method, implemented as a Matlab program. We used an adaptive Metropolis-Hastings (M-H) algorithm to carry out the MCMC procedure (see S1 Appendix B), and after a burn-in period of 500000 iterations the next 1000000 samplers gives estimates [6]. We estimated mean values including 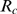 and other parameters and their stand deviations based on the exponential growth phase of hospital notifications between September 3rd and September 21st, the duration of exponential growth in the early stage of the outbreak. Our estimates of the reproduction number includes the effect of hospitalization of infectious cases as reported in data, and we therefore considered a parameter for isolation of diagnosed infectious cases.

To implement *Fengxiao* based on the meta-population model [9], we generated the dispersal rates among all patches independently and identically distributed (i.i.d.) on the interval 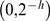, say, let 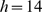 represent the *Fengxiao*, and 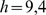 describe weak and strong dispersal, respectively. Define the upper and low threshold of the hospital notifications and switch on and off the *Fengxiao* measures together with or without strengthening local interventions in any university/college in terms of hospital notifications (S1 Appendix D). That is, the dispersal rates or local quarantine and precaution rates are updated when the hospital notifications in any community reaches the upper or low threshold. In simulating impact of nationwide travel on A/H1N1 flu outbreak in mainland China we changed dispersal rates and interventions in the fixed period of Spring Festival holiday on the basis of models 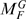 and 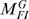.

## Supporting Information

Figure S1 Flow diagram for the pandemic H1N1 spread in mainland China, that incorporates disease progression and key intervention measures.
(0.01 MB EPS)

Figure S2 Estimates of the control reproduction number R_c_ in the province of Shaanxi during the wave of pandemic A/H1N1 influenza in the autumn of 2009. Estimates were generated with the use of MCMC and on the basis of (A) model M_F_, the mean R_c_ = 1.682; (B) the reduced model M_R_, the mean _c_ = 1.679; (C) the reduced model with asymptomatic infection M_RA_, the mean R_c_ = 1.792.
(0.02 MB EPS)

Figure S3 The best-fit solution obtained by fitting H(t) in model M_R_ to the hospital notifications from September 3rd to 21st, 2009.
(0.01 MB EPS)

Figure S4 Density estimates of posterior densities of five parameters and five initial values on the basis of model M_R_.
(0.02 MB EPS)

Figure S5 Sensitivity of relative infectiousness of the pre-symptomatic class (ε) and isolation rate (δ_3_) on model parameters. Sensitivity analyses for the effect of durations of latency and infectiousness before the onset of symptoms, and duration of infectiousness following symptoms onset on epsilon (A, B, C); on isolation rate (D, E, F), on the basis of model M_R_.
(0.02 MB EPS)

Table S1 Parameter definitions and values for the baseline model S1. Values for ρ, varrho and q_a_ are estimated by the model M_RA_. The mean value for β is calculated from the formula of R_c_ and the given ϕ. We choose q_e_∈[0, 0.6], q_p_ [0, 0.6] and ϕ∈ [0, 0.6] in our numerical simulations to represent variation of control strategies.
(0.03 MB PDF)

Table S2 Sensitivity analysis of mean R_c_ on the periods for model M_F_.
(0.02 MB PDF)

File S1 (0.13 MB PDF)
