# Supplementary material for: Community-Based Measures for Mitigating the 2009 H1N1 Pandemic in China
Source: PLoS One. 2010 Jun 18;5(6):e10911. doi: 10.1371/journal.pone.0010911 (PMC2887838; doi:10.1371/journal.pone.0010911)
Supplement: Table S1 — Parameter definitions and values for the baseline model S1. Values for ρ, varrho and qa are estimated by the model MRA. The mean value for β is calculated from the formula of Rc and the given ϕ. We choose qe∈[0, 0.6], qp [0, 0.6] and ϕ∈ [0, 0.6] in our numerical simulations to represent variation of control strategies. (0.03 MB PDF) [file pone.0010911.s006.pdf]

Parameter	Definition	Source	mean values
β	baseline transmission rate of infection	calculated	4.182
q_e	quarantine rate during latency infection (days^{-1})	MCMC	0.125
q_p	quarantine rate during pre-symptomatic infection(days^{-1})	MCMC	0.387
q_a	quarantine rate during asymptomatic infection(days^{-1})	MCMC	0.498
ϕ	proportion of exposed person who performed effective precaution ([0, 1])	assumed	0.4
ϵ	relative infectiousness of the pre-symptomatic class ([0, 1])	MCMC	0.515
ϱ	relative infectiousness of the asymptomatic class([0, 1])	MCMC	0.433
ρ	proportion of clinical infections ([0, 1])	MCMC	0.802
δ_1	rate of progression to pre-symptomatic class (days^{-1})	Tuite et al (2009)	1/2.62 (1/3.12-1/2.28)
δ_2	rate of progression to infectious with symptoms (days^{-1})	Tuite et al (2009)	1 (1/1.72-1/0.88)
δ_3	isolation rate (days^{-1})	MCMC	1.094
γ_1	recovery rate for symptomatic class (days^{-1})	Tuite et al (2009)	1/3.38 (1/4.69-1/2.06)
γ_2	recovery rate for hospitalized class (days^{-1})	Tuite et al (2009)	1/3.38 (1/4.69-1/2.06)
γ_3	recovery rate for asymptomatic class (days^{-1})	Gojovic et al.(2009)	1/2.5
