# Supplementary material for: Community-Based Measures for Mitigating the 2009 H1N1 Pandemic in China
Source: PLoS One. 2010 Jun 18;5(6):e10911. doi: 10.1371/journal.pone.0010911 (PMC2887838; doi:10.1371/journal.pone.0010911)
Supplement: Table S2 — Sensitivity analysis of mean Rc on the periods for model MF. (0.02 MB PDF) [file pone.0010911.s007.pdf]

from	to	mean R_c	95% CI
Sep.3	Sep.19	1.474	(1.260, 1.688)
Sep.3	Sep.20	1.626	(1.386, 1.866)
Sep.3	Sep.21	1.682	(1.446, 1.918)
Sep.3	Sep.22	1.644	(1.412, 1.876)
Sep.3	Sep.23	1.568	(1.376, 1.760)
Sep.3	Sep.24	1.476	(1.306, 1.646)
