# Supplementary material for: Community-Based Measures for Mitigating the 2009 H1N1 Pandemic in China
Source: PLoS One. 2010 Jun 18;5(6):e10911. doi: 10.1371/journal.pone.0010911 (PMC2887838; doi:10.1371/journal.pone.0010911)
Supplement: File S1 — (0.13 MB PDF) [file pone.0010911.s008.pdf]

Supporting Information (SI): Community-based measures for mitigating the 2009 H1N1 pandemic in China

In this supplementary material, we provide more detailed descriptions of *Fengxiao* strategy and the model equations, the calculation of the control reproduction number R_c, parameter determinations/estimations and numerical integration methods.

Fengxiao Since the emergence of influenza A/H1N1 pandemic virus in March-April 2009, mainland China quickly took a set of very strict nonpharmaceutical interventions (NPIs): intensive contact tracing followed by quarantine of suspected individuals who have the high risk of having been exposed to the virus and by isolation of symptomatic individuals; closure of schools, and other measures. The province of Shaanxi, for example, introduced a series of measures including quarantine and isolation aiming at controlling university cases as a response to the situation where most cases in early September were associated with schools and university/college campuses. *Fengxiao*, a special proactive measure, was fiercely implemented. This measure prohibits college and university students, faculty and staff members to leave their campuses, disallows visit to the campus of non college/university members, and carefully monitors the essential service providers who have to cross the campus boundaries, while campus normal activities being kept. Such a measure was possible in mainland China since the vast majority of college students have their residency on campus and college faculty and staff members are housed in campus special zones. Complete isolation was of course impossible, but *Fengxiao* seemed to have reduced the transmission from the campuses into the wider community (*Fengxiao*, 2009).

Appendix A: Baseline Models and Control Reproduction Number

The full model M_F We formulated a baseline model that reflects some key epidemiological properties of the pandemic H1N1 influenza and the implemented public health interventions (quarantine, isolation and hygiene precaution). We assume a susceptible individual exposed to the virus may or may not become infected, depending on the protection measures adopted by the individual. Once infected, the individual moves to the early latent compartment E_1, the early stage of being infected but not yet infectious. This stage is then followed by a presymptomatic infectious period (with the corresponding compartment denoted by E_2 (Gojovic et al., 2009)). The individual passing through this presymptomatic period will continue to be infectious (with different infectioness), either asymptomatically (A) or symptomatically (I), until recovery from or die of the disease

(R). Some of the infectious individuals will be hospitalized (H). Using the standard incidence and following the flow diagram in Figure S1, we have the following baseline compartmental model

$$\begin{cases} S' &= -\frac{(1-\phi)(\beta I+\epsilon\beta E_2)S}{N}, \\ E_1' &= \frac{(1-\phi)(\beta I+\epsilon\beta E_2)S}{N} - (\delta_1 + q_e)E_1, \\ E_2' &= \delta_1 E_1 - (\delta_2 + q_p)E_2, \\ I' &= \delta_2 E_2 - (\delta_3 + \gamma_1)I, \\ Q_{E_1}' &= q_e E_1 - \delta_1 Q_{E_1}, \\ Q_{E_2}' &= q_p E_2 + \delta_1 Q_{E_1} - \delta_2 Q_{E_2}, \\ H' &= \delta_2 Q_{E_2} + \delta_3 I - \gamma_2 H, \\ R' &= \gamma_1 I + \gamma_2 H, \end{cases} \tag{S1}$$

where $'$ is the derivative with respect to time t, $N = S + E_1 + E_2 + I + Q_{E_1} + Q_{E_2} + H + R$ is the total population which is assumed to be a constant. When contact tracing and quarantine are implemented, the parameters q_e and q_p model the rate of quarantining infected but not yet infectious and infectious pre-symptomatic individuals respectively. These individuals move to the compartment Q_{E1} and Q_{E2} respectively. Those in the Q_{E1} class then progress to the Q_{E2} and will be hospitalized once they develop symptoms. Our model also incorporates precautionary measures: when effective precautionary measures are taken, a proportion, ϕ of the individuals exposed to the virus is protected from the infection. ϵ is the relative transmissibility of pre-symptomatic infection. The definitions and evaluations of other parameters for the model (S1) are listed in Table S1.

We calculated the basic reproduction number by using the next generation matrix (Diekmann and Heesterbeek , 2000; Van den Driessche and Watmough, 2002). In the main text, we call this control reproduction number since interventions were implemented during the period of consideration. This reproduction number R_c, the spectral radius of the next generation matrix FV^{-1}, is given by

$$R_c = \rho(FV^{-1}) = \frac{\delta_1(1-\phi)}{(\delta_1 + q_e)(\delta_2 + q_p)}\Big(\frac{\beta\delta_2}{\delta_3 + \gamma_1} + \epsilon\beta\Big), \tag{S2}$$

where

$$F = \begin{pmatrix} 0 & \epsilon\beta(1-\phi) & \beta(1-\phi) \\ 0 & 0 & 0 \\ 0 & 0 & 0 \end{pmatrix}, \quad V = \begin{pmatrix} \delta_1 + q_e & 0 & 0 \\ -\delta_1 & \delta_2 + q_p & 0 \\ 0 & -\delta_2 & \delta_3 + \gamma_1 \end{pmatrix}.$$

Note that each term of the aforementioned expression for R_c has clear epidemiological interpretation. A fraction $\delta_1/(\delta_1 + q_e)$ goes from E_1 to the infectious but not yet symptomatic (pre-symptomatic) class E_2, with the controlled contact rate $\epsilon\beta(1-\phi)$ and mean

duration $1/(\delta_2 + q_p)$, giving a contribution of $\epsilon\beta(1-\phi)\delta_1/(\delta_1 + q_e)(\delta_2 + q_p)$. A fraction $\delta_1\delta_2/(\delta_1 + q_e)(\delta_2 + q_p)$ goes from E_1 to the infectious and symptomatic class I, with the controlled contact rate $\beta(1-\phi)$ and mean duration $1/(\delta_3 + \gamma_1)$, giving a contribution of $\beta(1-\phi)\delta_1\delta_2/(\delta_1 + q_e)(\delta_2 + q_p)(\delta_3 + \gamma_1)$. The sum of these individual contributions gives R_c.

In order to directly estimate R_c, we introduce the aggregated parameter

$$P_0 \triangleq \frac{\delta_1}{(\delta_1 + q_e)(\delta_2 + q_p)}\left(\frac{\delta_2}{\delta_3 + \gamma_1} + \epsilon\right).$$

As such, the mode (S1) becomes

$$\begin{cases} S' &= -\left(\frac{R_c}{P_0}\frac{SI}{N} + \frac{\epsilon R_c}{P_0}\frac{SE_2}{N}\right), \\ E_1' &= \frac{R_c}{P_0}\frac{SI}{N} + \frac{\epsilon R_c}{P_0}\frac{SE_2}{N} - (\delta_1 + q_e)E_1, \\ E_2' &= \delta_1 E_1 - (\delta_2 + q_p)E_2, \\ I' &= \delta_2 E_2 - (\delta_3 + \gamma_1)I, \\ Q_{E_1}' &= q_e E_1 - \delta_1 Q_{E_1}, \\ Q_{E_2}' &= q_p E_2 + \delta_1 Q_{E_1} - \delta_2 Q_{E_2}, \\ H' &= \delta_2 Q_{E_2} + \delta_3 I - \gamma_2 H, \\ R' &= \gamma_1 I + \gamma_2 H. \end{cases} \tag{S3}$$

This shows that the parameter $\beta(1-\phi)$ can be determined by the estimates of $q_e, q_p, \delta_3, \epsilon$ and R_c.

Simplified model M_R during the initial growth stage To estimate the mean R_c and its standard deviation without estimating the initial susceptible population size, and to reduce the number of parameters that needed to be estimated to the minimal based on hospital notifications (laboratory-confirmed cases) during the initial exponential growth, we note that in the early outbreak S/N approximately equals to 1 and hence we have the following reduced model, denoted by M_R

$$\begin{cases} E_1' &= \frac{R_c}{P_0}I + \frac{\epsilon R_c}{P_0}E_2 - (\delta_1 + q_e)E_1, \\ E_2' &= \delta_1 E_1 - (\delta_2 + q_p)E_2, \\ I' &= \delta_2 E_2 - (\delta_3 + \gamma_1)I, \\ Q_{E_1}' &= q_e E_1 - \delta_1 Q_{E_1}, \\ Q_{E_2}' &= q_p E_2 + \delta_1 Q_{E_1} - \delta_2 Q_{E_2}, \\ H' &= \delta_2 Q_{E_2} + \delta_3 I - \gamma_2 H. \end{cases} \tag{S4}$$

Simplified model M_{RA} with asymptomatic infection Adding the asymptomatic infection class to the above simplified model enables us to evaluate the impact of asymptomatic infection on the estimation of R_c and the disease growth potential. A fraction ρ goes from

E_2 to the asymptomatic class A. Let ϱ be the relative infectiousness of asymptomatic infection, $1/\gamma_3$ be the mean duration of asymptomatic infection. This yields the following

$$\begin{cases} E_1' &= \frac{R_c}{P_1}I + \frac{\epsilon R_c}{P_1}E_2 + \frac{\varrho R_c}{P_1}A - (\delta_1 + q_e)E_1, \\ E_2' &= \delta_1 E_1 - (\delta_2 + q_p)E_2, \\ I' &= (1-\rho)\delta_2 E_2 - (\delta_3 + \gamma_1)I, \\ A' &= \rho\delta_2 E_2 - (q_a + \gamma_3)A, \\ Q_{E_1}' &= q_e E_1 - \delta_1 Q_{E_1}, \\ Q_{E_2}' &= q_p E_2 + \delta_1 Q_{E_1} - \delta_2 Q_{E_2}, \\ H' &= \delta_2 Q_{E_2} + \delta_3 I - \gamma_2 H \end{cases} \tag{S5}$$

with the reproduction number

$$R_c = \frac{\delta_1(1-\phi)}{(\delta_1 + q_e)(\delta_2 + q_p)}\left(\frac{(1-\rho)\beta\delta_2}{\delta_3 + \gamma_1} + \epsilon\beta + \frac{\varrho\beta\rho\delta_2}{\gamma_3 + q_a}\right), \tag{S6}$$

and

$$P_1 = \frac{\delta_1}{(\delta_1 + q_e)(\delta_2 + q_p)}\left(\frac{(1-\rho)\delta_2}{\delta_3 + \gamma_1} + \epsilon + \frac{\varrho\rho\delta_2}{\gamma_3 + q_a}\right).$$

Appendix B: Parameter Estimation

The irregularity of the data used is caused by the lack of information over the weekends and the October National Day holidays, and the change of reporting policy. Therefore, we used the cubic spline interpolation method to generate the daily number of hospital notifications.

The variance of measured component, $H(t)$, was given by inverse gamma distribution with hyper-parameters (0.01, 4), where 0.01 is the initial error variance which was updated by inverse gamma distribution (see http://www.helsinki.fi/ mjlaine/mcmc/), and the small MCMC package provided in this website was used to estimate the parameters. When estimating unknown parameters and initial values for models M_F, M_R and M_{RA}, the following prior informations were given: $R_c \in (1, 5), q_e \in (0, 0.5), q_p \in (0, 1), q_a \in (0, 1), \epsilon \in (0, 1)$, $\rho \in (0, 1), \varrho \in (0, 1)$, $\delta_3 \in (0.5, 2)$, $S(0) \in (10^5, 10^6), E_1(0) \in (0, 100)$, $E_2(0) \in (0, 100), I(0) \in (0, 100), Q_{E_1}(0) \in (0, 100), Q_{E_2}(0) \in (0, 100)$, and the proposal density was chosen to be a multivariate normal distribution. These priors were used to ensure good convergence of the MCMC chain.

The joint posterior distribution of the parameters and initial values were explored via MCMC sampling. Here we used adaptive Metropolis-Hastings algorithm to carry out the MCMC procedure (Haario, 2006). The algorithm runs for 1000000 iterations with a burn-in of 500000 iterations, and the Geweke convergence diagnostic method was employed

to assess convergence of chains (Geweke, 1992). Convergence can be evaluated by the proximity of Geweke value to 1.

For the data from the province of Shaanxi, we note that there were 21 imported (from overseas or other provinces) laboratory-confirmed cases before September 3rd. These cases did not cause secondary infections and recovered by the end of August (See http://www.sxhealth.gov.cn/h1n1.asp). Therefore, these 21 laboratory-confirmed cases were not included in our parameter estimation.

The estimation of the reproduction number R_c depends on the period under consideration. To show the sensitivity of such a reproduction on the period considered, we considered the periods of Sep 3-19, 3-20, 3-21, 3-22, 3-23, and 3-24, and our results are presented in Table S2 below.

Appendix C: Metapopulation Models

We then followed (Levin, 1974) and extend our baseline model to a metapopulation, where coupling among patches is through dispersal on a dispersal network. We used this model framework in two different settings: the spread among a network of universities/colleages within a city (Xi'an) and the spread among the national network of provinces. In each patch, the progression of the disease is tracked by defining the disease states similar to the baseline model M_F. Transmission among patches is represented by dispersal of individuals via the dispersal networks. The purpose is to evaluate the effectiveness and interactions of different spatially relevant interventions: *Fengxiao*, quarantine, precaution, dispersal and transport-related infection control.

Patch Model M_F^G The general patch model extended from our baseline model M_F is as follows

$$\left\{\begin{array}{rcl} S_i' & = & -\frac{(1-\phi_i)(\beta I_i+\epsilon\beta E_{2i})S_i}{N_i} + \sum\limits_{j=1}^{n} S_j d_{S_{ij}} D_{ij}, \\ E_{1i}' & = & \frac{(1-\phi_i)(\beta I_i+\epsilon\beta E_{2i})S_i}{N_i} - (\delta_1 + q_{ei})E_{1i} + \sum\limits_{j=1}^{n} E_{1j} d_{E_{1ij}} D_{ij}, \\ E_{2i}' & = & \delta_1 E_{1i} - (\delta_2 + q_{pi})E_{2i} + \sum\limits_{j=1}^{n} E_{2j} d_{E_{2ij}} D_{ij}, \\ I_i' & = & \delta_2 E_{2i} - (\delta_3 + \gamma_1)I_i + \sum\limits_{j=1}^{n} I_j d_{I_{ij}} D_{ij}, \\ Q_{E_{1i}}' & = & q_{ei}E_{1i} - \delta_1 Q_{E_{1i}}, \\ Q_{E_{2i}}' & = & q_{pi}E_{2i} + \delta_1 Q_{E_1 i} - \delta_2 Q_{E_{2i}}, \\ H_i' & = & \delta_2 Q_{E_{2i}} + \delta_3 I_i - \gamma_2 H_i, \\ R_i' & = & \gamma_1 I_i + \gamma_2 H_i + \sum\limits_{j=1}^{n} R_j d_{R_{ij}} D_{ij}, \end{array}\right. \tag{S7}$$

where $i = 1, 2, \cdots, n$. Note that N_i, the population size in patch i, is not a constant but $\sum\limits_{i=1}^{n} N_i$ is assumed to be a constant. Dispersal among patches is governed by the dispersal

rates d_S (susceptibles), d_E (incubation), d_I (infectives) and d_R (removed) and the matrix D. The matrix $D = G - M$ represents the allowed dispersal transitions, where G is the adjacency matrix of the dispersal network, meaning that $G_{ij} = 1$ if individuals are allowed to move from patch j to patch i. M represents emigration, and is thus a diagonal matrix with entries $M_{ii} = \sum_{j=1}^{n} G_{ji}$. Here we have neglected the death rates and birth rates of individuals during the dispersal process, i.e. we have

$$\sum_{i=1}^{n} d_{S_{ij}} D_{ij} = 0, \sum_{i=1}^{n} d_{E_{1ij}} D_{ij} = 0, \sum_{i=1}^{n} d_{E_{2ij}} D_{ij} = 0, \sum_{i=1}^{n} d_{I_{ij}} D_{ij} = 0, \sum_{i=1}^{n} d_{R_{ij}} D_{ij} = 0,$$

for all $j = 1, 2, \cdots, n$. The small-world network introduced by Watts and Strogatz (1998) is employed to generate matrix G with an average number of connections per vertex (degree) of four and rewiring each connection with probability p ($p = 0.2$ is chosen in the main text).

Model M_{FI}^{G} with Transport-Related Infection To investigate the impact of infection during travel on the pandemic trend nationwide, we included transport-related infection to the patch model M_F^G and obtained

$$\left\{\begin{array}{rcl}
S_i' & = & -\frac{(1-\phi_i)(\beta I_i+\epsilon\beta E_{2i})S_i}{N_i} + \sum\limits_{j=1}^{n} S_j d_{S_{ij}} D_{ij} - FI_i, \\
E_{1i}' & = & \frac{(1-\phi_i)(\beta I_i+\epsilon\beta E_{2i})S_i}{N_i} - (\delta_1 + q_{ei})E_{1i} + \sum\limits_{j=1}^{n} E_{1j} d_{E_{1ij}} D_{ij} + FI_i, \\
E_{2i}' & = & \delta_1 E_{1i} - (\delta_2 + q_{pi})E_{2i} + \sum\limits_{j=1}^{n} E_{2j} d_{E_{2ij}} D_{ij}, \\
I_i' & = & \delta_2 E_{2i} - (\delta_3 + \gamma_1)I_i + \sum\limits_{j=1}^{n} I_j d_{I_{ij}} D_{ij}, \\
Q_{E_{1i}}' & = & q_{ei}E_{1i} - \delta_1 Q_{E_{1i}}, \\
Q_{E_{2i}}' & = & q_{pi}E_{2i} + \delta_1 Q_{E_1 i} - \delta_2 Q_{E_{2i}}, \\
H_i' & = & \delta_2 Q_{E_{2i}} + \delta_3 I_i - \gamma_2 H_i, \\
R_i' & = & \gamma_1 I_i + \gamma_2 H_i + \sum\limits_{j=1}^{n} R_j d_{R_{ij}} D_{ij},
\end{array}\right. \tag{S8}$$

where

$$FI_i = \sum_{j\neq i, j=1}^{n} \frac{\eta D_{ij} d_{S_{ij}} S_j \left(d_{I_{ij}} I_j + \epsilon d_{E_{2ij}} E_{2ij}\right)}{d_{S_{ij}} S_j + d_{I_{ij}} I_j + d_{E_{1ij}} E_{1ij} + d_{E_{2ij}} E_{2ij} + d_{R_{ij}} R_{ij}},$$

η is the transport-related transmission rate. In our simulations, we used $\eta = 0, \eta = 2.5$ and $\eta = 3.75$ to denote no infection, weak infection and strong infection during travel, respectively.

Appendix D: Numerical Integration for Models M_F^G and M_{FI}^G

Numerical integrations for the network models M_F^G and M_{FI}^G were carried out using the Runge-Kutta method in Matlab 7.0. All simulations were initiated with pseudorandomly

generated dispersal rates, with $d_{S_{ij}}, d_{E_{1ij}}, d_{E_{2ij}}, d_{I_{ij}}, d_{R_{ij}}$ independently and identically distributed (i.i.d.) among all patches on the interval $(0, 2^{-h})$. *Fengxiao* was not complete isolation of a university/college, so we chose the dispersal rate sufficiently small to describe *Fengxiao*. In our simulation analysis of the *Fengxiao* strategy based on the metapopulation model, we randomly generated the dispersal rates among communities from the interval $(0, 2^{-14})$ to represent the *Fengxiao*, from the interval $(0, 2^{-9})((0, 2^{-4}))$ to describe weak (strong) dispersal.

Numerical integration for *Fengxiao***(Figure 4)** To integrate the model M_F^G, we assumed that there are $n = 51$ patches, which is the total number of colleges and universities in Xi'an, the capital city of the province of Shaanxi (See http://www.edu.cn). The parameter values and initial values are listed in Table 1 of the main text and Table S1. Note that we initially let $q_e = 0, q_p = 0$ and $\phi = 0$ to examine effects of *Fengxiao* on the spread among the college network of the city. We allocated the estimated total susceptible population of the province of Shaanxi to 51 universities/colleges (that is, we assumed that there were on average 555310/51 individuals in each of these colleges and universities). The first cluster of confirmed cases was reported in Xi'an Institute of Arts and Sciences where there were nearly ten thousand full-time students and more than 900 faculty members. The Institute implemented *Fengxiao* on September 4, 2009 (See http://www.sxhealth.gov.cn/h1n1.asp). We randomly chose one of 51 patches and set the initial values to be the values estimated and listed in Table 1 of the main text with $S(0) = 555310/51$. For all the other patches we set the initial values as $(555310/51, 0, 0, 0, 0, 0, 0, 0, 0)$.

To simulate the model M_F^G with a variety of interventions, we initially set $h = 4$. Once the daily hospital notifications in patch i reached a given threshold value $H^{\max}$, we implemented *Fengxiao* in patch i which was realized by increasing h from 4 to 14 so the dispersal rate from the i-th patch to all other patches and the dispersal rate from all other patches to the i-th patch are reduced. Alternatively, we simulated the model by strengthening the local control measures (increasing quarantine and precaution, q_{ei}, q_{pi} and ϕ_i). When the daily hospital notifications in the $i-$th patch decreased to the low threshold value $H^{\min}$, we suspended *Fengxiao* by changing h from 14 back to 4, and/or relaxing the other local control strategies by decreasing quarantine parameters q_{ei}, q_{pi} and precaution parameter ϕ_i.

Several scenarios were considered, as described below.

Case 1: *Fengxiao* alone. In this case, only the dispersal rates were reduced when the hospital notifications reached the threshold value $H^{\max}$. When the hospital notifications decreased and reached the threshold value $H^{\min}$, the dispersal rates returned back to the original values.

Case 2: Local control measures without *Fengxiao*. In this case, we increased q_{ej},

q_{pj} and ϕ_j from 0 to 0.6 when the hospital notifications reached the $H^{\max}$. When the hospital notifications decreased and reached the threshold value $H^{\min}$, the local control policy changed according to following rues

a. Changed the values of q_{ej}, q_{pj} and ϕ_j to the baseline values (0.125, 0.387 and 0.4);

b. Changed the values of q_{ej}, q_{pj} and ϕ_j to 0.4.

Case 3: Combination local control strategies and *Fengxiao*. In this case, we increased q_{ej}, q_{pj} and ϕ_j from 0 to 0.6 and applied *Fengxiao* when the hospital notifications reached the $H^{\max}$. When the hospital notifications decreased to the threshold value $H^{\min}$, *Fengxiao* was suspended, and the local control measures changed according to following

a. Changed the values of q_{ej}, q_{pj} and ϕ_j to the baseline values (0.125, 0.387 and 0.4);

b. Changed the values of q_{ej}, q_{pj} and ϕ_j to 0.4.

Numerical integration for Figures 5 and 7 in the main text

Case 4: To address the effect of nationwide travel during the Spring Festival, we considered a 32-patch system (including 23 provinces, 4 municipalities and 5 autonomous regions in mainland China) and used parameter values from the Province of Shaanxi as the baseline parameters for each patch (Table 1 in main text) except the values of q_{ei}, q_{pi} and ϕ_i which were taken as $q_{ei} = q_{pi} = \phi_i = 0.4$. The initial values for all patches were generated by Poisson distribution with mean values coming from the Shaanxi data listed in Table 1 of the main text. Initially, the weak dispersal rates were generated for Figures 4 and 6 and the low transmission during travel ($\eta = 2.5$, which equals to the value of $\beta(1-\phi_i)$) was chosen for Figure 6. Simulating models M_F^G and M_{FI}^G gave Figures 5(A-B) and 7(A-B) from May 11 2009 when the first imported case was confirmed in mainland China, respectively.

Figure 5(C-H) showed the outcome with some changes of interventions from February 1st to March 1st 2010: the dispersal rates were increased from 2^{-9} to 2^{-4} in Figure 5(C-D); the susceptible population sizes were doubled in Figure 5(E-F); and the quarantine rates were reduced by 20% in Figure 5(G-H).

Figure 7(C-H) illustrated the impact of travel-related changes from February 1st to March 1st 2010: the dispersal rates were increased from 2^{-9} to 2^{-4} Figure 7(C-D); the transmission rate during travel was increased to $\eta = 3.75$ in Figure 7(E-F); both dispersal rates and transmission during travel were increased in Figure 7(G-H).

References

Fengxiao strategy (2009). http://www.moe.edu.cn/edoas/web- site18/level3.jsp? tablename=603&infoid=1259 567757094146.

Diekmann O, Heesterbeek JAP (2000). *Mathematical epidemiology of infectious diseases: model building, analysis and interpretation* (Chichester: John Wiley).

Geweke J (1992). *Evaluating the Accuracy of Sampling-Based Approaches to the Calculation of Posterior Moments*. In Bayesian Statistics 4 (eds. J.M. Bernardo, J. Berger, A.P. Dawid and A.F.M. Smith), Oxford: Oxford University Press, 169-193.

Gojovic MZ et al. (2009). Modelling mitigation strategies for pandemic (H1N1) 2009. *CAMJ 2009* DOI:10.1503/cmaj.091641.

Haario H, Laine M, Mira A, Saksman E (2006). DRAM: Efficient adaptive MCMC. *Statistics and Computing* 16:339-354.

Levin SA (1974). Dispersion and population interactions. *Amer Natur*108:207-228.

Tuite AR et al. (2009). Estimated epidemiologic pearameters and morbidity associated with pandemic H1N1 influenza. *CMAJ 2009* DOI:10.1503/cmaj.091807.

Watts DJ, Strogatz SH (1998). Collective dynamics of 'small-world' networks. Nature 393:440-442.

Van den Driessche P, Watmough J(2002) Reproduction numbers and sub-threshold endemic equilibria for compartmental models of disease transmission. *Math Biosci* 180:29-48.
